# Relationship Between Affective Temperaments and Suicide Risk in Patients With First-Onset Major Depressive Disorder

**DOI:** 10.3389/fpsyt.2022.893195

**Published:** 2022-06-06

**Authors:** Lu Yin, Tian-He Song, Yan-Yan Wei, Li-Gang Zhang, Shuang-Jiang Zhou, Jian-Jin Yu, Li-Ye Zhang, Hong-Juan Li, Jing-Xu Chen

**Affiliations:** ^1^Beijing Hui-Long-Guan Hospital, Peking University Hui-Long-Guan Clinical Medical School, Beijing, China; ^2^Department of Psychology, Chengde Medical University, Hebei, China

**Keywords:** suicide risk, temperament, major depressive disorder, first-onset, TEMPS-A

## Abstract

**Background:**

People may endorse suicidal behavior during a major depressive episode. Affective temperaments may play a role in this risk. We explored the relationship between affective temperaments and suicide and identified some traits that can predict suicide risk in depression.

**Materials and Methods:**

We analyzed the results of the Temperament Evaluation of the Memphis, Pisa, Paris, and San Diego Auto-questionnaire (TEMPS-A) in 284 participants recruited from a psychiatric clinic and the community in Beijing and compared the subscale scores (temperaments of cyclothymic, dysthymic, anxious, irritable, and hyperthymic) among major depressive disorders (MDDs) vs. the general population as well as depressive patients with vs. without suicide risk, using Student’s test, chi-square test, rank-sum test, and multivariable regression modeling.

**Results:**

The incidence of suicidal risk in depressive subjects was 47.62% (80/168). Being unmarried (*p* < 0.001), unemployed (*p* = 0.007), and temperaments of dysthymic, cyclothymic, anxious, and irritable scores (all *p* < 0.001) were significantly more prevalent in patients with depression than in the general population. Young age (*p* < 0.001), female sex (*p* = 0.037), unmarried (*p* = 0.001), more severe depression (*p* < 0.001), and dysthymic, anxious, and cyclothymic temperament (all *p* < 0.05) were significantly more prevalent in patients with depressive disorder than those without suicide risk. The logistic regression analysis showed that younger age (odds ratio [*OR*] = 0.937, 95% *CI* 0.905∼0.970), female sex (*OR* = 2.606, 95% *CI* 1.142∼5.948), more severe depression (*OR* = 1.145, 95% *CI* 1.063∼1.234), cyclothymic temperament (*OR* = 1.275, 95% *CI* 1.102∼1.475), and dysthymic temperament (*OR* = 1.265, 95% *CI* 1.037∼1.542) were all independently associated with high suicidal risk in patients with first-onset major depression (*p* < 0.05).

**Conclusion:**

Temperament traits differ between the general population and people suffering from MDD. Subjects with MDD who have much more severe depressive symptoms and a cyclothymic or dysthymic temperament were at a high risk of suicide.

## Introduction

Suicide, the act of intentionally injuring oneself with the intent of dying, including attempt, preparation, ideation, and planning, is a challenging and complex public health problem that affects all countries (1). According to the WHO website data, close to 703,000 people died by suicide worldwide in 2019 (2). The latest data from the United States referenced 47,511 people dying of suicide in 2019 (3). Meanwhile, that number in China was approximately 126,000 people and ranked 13 in all-cause mortality in 2017 (4). Suicide can occur at any time of life, but in young people aged 15–29, it ranks fourth among causes of death in a report from 2019 (2). There are many reasons people commit suicide, such as physical diseases, mental disorders, life stressors, or events. Studies on factors leading to suicide have been undertaken for decades. The dimensions include but are not limited to genes, neuroanatomy, biochemistry, the environment, nutrition, physical and mental diseases, mainly depression, and alcohol abuse (2, 5–9), but suicide prevention remains an area of uncertainty and needs further research (10).

According to a cross-sectional epidemiological nationwide study conducted during 2013–2015 in China, the lifetime prevalence of mood disorders was 7.4% (4). People with mood disorders’ lifetime prevalence of suicidal ideation, planning, and attempts were 53.1, 17.5, and 23.7%, respectively (11), which were much higher than those in the Chinese general population (3.1, 0.9, and 1.0%, respectively) (12). Those study results make mood disorder an unignorable factor for suicide prevention.

Temperament describes individual differences in behavior based on biology (13). It derives from the original theory of Hippocrates and Galen about chemical imbalance and modern studies of neurochemistry and psychiatry (13). Temperament traits are diverse among different populations, and there have been some independent studies on depressive patients and the general population (14–16), but there is still a lack of comparative studies between the two kinds of populations.

In China, the majority of patients with first-episode depression will present to primary care, with problems other than mood disorder (17). As there is a low recognition rate, many patients with depression commit suicide before they seek treatment at psychiatric clinics, which still lack data but provide a lot of news. For suicide prevention, we need to find a relationship between suicide and temperament. There were many scales that could be chosen for our research purpose, for example, the Functional Ensemble of Temperament (FET) including emotional, physical, verbal, and mental aspects traits of behavior (18, 19). Since mood is the main clinical symptom of depressed patients, we mainly choose a scale measuring temperament only. Two studies evaluating participants from the general population and patients with depressive disorder assessed by the Temperament Evaluation of the Memphis, Pisa, Paris, and San Diego Auto-questionnaire (TEMPS-A) showed that cyclothymic, irritable, and especially dysthymic temperaments might be important factors in self-harm in adolescents (20). In patients with depression, cyclothymic and dysthymic temperaments were associated with suicide acts in bipolar disorder compared with major depressive disorder (MDD) (21). These studies are the basis of our further research on temperament traits in first-onset MDD. We know that temperament cannot solely predict suicide risk in neither the general population nor depressive disorders, but we hope to sketch features associated with suicide act and find some common characteristics as either a theoretical foundation or practical guidance for clinicians.

## Materials and Methods

### Subjects

All subjects were recruited from February 2018 to September 2019 in the outpatient clinic of Beijing HuiLongGuan Hospital, affiliated with the Clinical Medical College of Peking University, China. All participants provided written informed consent that was approved by the institutional review board of the Beijing HuiLongGuan Hospital, Peking University. The inclusion criteria were 18–60 years, Han ethnicity, first-onset of depression, educational level above junior middle school, and MDD diagnosed by the Diagnostic and Statistical Manual of Mental Disorders, Fifth Edition (DSM-5) on the consensus of two independent senior psychiatrists titled with associate chief physician or above. Patients were excluded if they had an organic mental disorder, substance use disorder, developmental delay, neurological illness, psychotic disorder, or were pregnant or breastfeeding.

Healthy controls (HCs) were enrolled from the community near the hospital according to the criteria of Han Chinese individuals without any personal or family history of mental illness. The exclusion criteria were the same as those for the patient group.

### Instruments

Major depressive disorder was diagnosed by the Chinese version of the Mini International Neuropsychiatric Interview (M.I.N.I.), Version 5.0 (22), a short-structured interview including six questions on suicidality, which is a useful tool for assessing suicide risk (23–25). The suicide risk section scores ranged from 0 to 33. As in our previous study, patients with a score <6 were classified as having non-suicidal risk, and those with a score ≥6 were classified as having suicidal risk (9), which includes suicide ideation, suicide act, or both. The 17-item Hamilton Depression Rating Scale (HAMD-17) was used to assess the severity of depressive symptoms (26). The third (suicidal) item was excluded when evaluating the association between suicidal risk and the severity of depression. Considering the rating consistency and reliability of our study, five psychiatrists attended a training session on the use of the M.I.N.I. and HAMD-17.

Affective temperaments were evaluated by the short version of the TEMPS-A (27, 28). The TEMPS-A is a self-report questionnaire containing 39 items measuring five affective temperament tendencies of people: dysthymia, cyclothymia, hyperthymia, anxiety, and irritation. Every subject received a “*Z*-score” on each of the five temperament dimensions. The dominant temperament is generated from the comparison of the five *Z*-scores. The range around one Z-score (Z1) of the mean (mean ± 1 SD) is considered the “standard” score, “mild deviation” is the interval between Z1 and Z2 (mean ± 2 SD), and “moderate deviation” is the Z-score equal to or above Z2 (mean ± 2 SD).

### Statistical Analysis

Data are presented as the means ± SDs, proportions, or medians. Preliminary tests of association used standard bivariate comparisons based on analysis of variance (ANOVA) methods or rank-sum tests for continuous data and contingency tables for categorical data. Multiple comparisons between suicidal and non-suicidal risk depressive patients (NSR-P) and HCs were tested by the Kruskal–Wallis *H*-test, and the resulting *p*-values were adjusted. The Mann–Whitney *U*-test was used for continuous variables (including the total score of temperament dimensions). All factors statistically significant in preliminary tests were put into logistic regression modeling, and the alpha level was set at 5%. Analyses were performed using the commercial software SPSS version 20.0.

## Results

### Demographic and Temperament Characteristics in Patients With Major Depressive Disorder and Healthy Controls

There were significant differences in marital status (odds ratio [*OR*] = 4.300, 95% *CI* 2.580∼7.166) and vocational status (*OR* = 3.144, 95% *CI* 1.311∼7.398) between patients with MDD and HCs (Table 1). Scores for both groups on dysthymic, cyclothymic, anxious, and irritable temperaments were significantly different (all *p* < 0.001). Compared with healthy people, patients with depression had higher scores on all temperaments except hyperthymic, especially on dysthymic (*U* = 1,859.50, *z* = 11.74) and cyclothymic temperaments (*U* = 2,728.00, *z* = 10.32) (Table 2). Scores of hyperthymic temperament for healthy individuals (mean rank = 136.40) and patients with first-episode MDD (mean rank = 146.71) were not significantly different (*p* = 0.290) by using the exact sampling distribution for *U* (Table 2).

**TABLE 1:** Comparisons of demographic characteristics between patients with MDD and HCs.

Variables	MDD (*N* = 168)	HCs (*N* = 116)	Statistic (*t* or *x*^2^)	OR	95% CI		*P*-value
					Lower	Upper	
Age (years), Mean (SD)	30.40 (12.50)	32.15 (9.46)	1.27	1.014	0.993	1.035	0.205
Sex, *n* (%)			0.01	1.031	0.630	1.685	0.904
Male	62 (36.90)	42 (36.21)					
Female	106 (63.10)	74 (63.79)					
Education, *n* (%)			0.39	0.847	0.502	1.429	0.533
High school and below (<12 years)	45 (26.79)	35 (30.17)					
Above high school (≥12 years)	123 (73.21)	81 (69.83)					
Marital status, *n* (%)			32.96	4.300	2.580	7.166	<0.001*
Married	62 (36.90)	83 (71.55)					
Unmarried	106 (63.10)	33 (28.45)					
Vocational status, *n* (%)			7.18	3.114	1.311	7.398	0.007*
On the job	140 (83.33)	109 (93.97)					
Unemployment	28 (16.67)	7 (6.03)					

*MDD, major depressive disorder; HCs, healthy controls.*

*Statistically significance.

**TABLE 2:** Comparisons of the temperament evaluation of the Memphis, Pisa, Paris, and San Diego Auto-questionnaire (TEMPS-A) between MDD and HCs.

Variables	MDD (*N* = 168)				HCs (*N* = 116)				Statistic U	Statistic z	*P*-value
	Mean rank	Median	95% CI		Mean rank	Median	95% CI				
Dysthymic temperament	189.43	5	4.26	4.93	74.53	0	0.61	1.20	1859.50	11.74	<0.001*
Cyclothymic temperament	184.07	7	6.40	7.30	82.29	1	1.80	2.85	2728.00	10.32	<0.001*
Anxious temperament	177.59	2	1.55	1.90	91.68	0	0.32	0.59	3849.00	9.06	<0.001*
Irritable temperament	163.70	2	1.64	2.18	111.79	0	0.62	1.10	6182.00	5.46	<0.001*
Hyperthymic temperament	146.71	2	2.08	2.73	136.40	2	1.79	2.61	9036.00	1.06	0.290

*MDD, major depressive disorder; HCs, healthy controls.*

*Statistically significant.

### Demographic and Temperament Characteristics of Suicidal and Non-suicidal Risk in Patients With Major Depressive Disorder

Comparing the differences between suicidal and non-suicidal subgroups of the patients with first-onset MDD, the younger the current age, the higher the suicidal risk (*OR* = 1.070, 95% *CI* 1.037∼1.104). Suicidal risk depressive patients (SR-P) saw doctors at an age of 25.79 ± 10.51 years, and the NSR-P saw doctors at an age of 34.6 ± 10.51 years. Women were statistically significant at higher risk of suicide than men (*OR* = 0.507, 95% *CI* 0.267∼0.963), and the constituent ratio of women in the suicide group was 71.25%. Unmarried people (76.25%) are statistically significant at higher suicidal risk than married people (*OR* = 3.068, 95% *CI* 1.581∼5.955). People with higher HAMD scores were statistically significant than those with lower scores at higher suicidal risk (*OR* = 0.873, 95% *CI* 0.823∼0.925). However, employment and educational level were not statistically significant risk factors for suicide in first-onset MDD. The median illness durations of suicidal and non-suicidal patients with MDD were 6.5 and 6 months, respectively, and the differences were not statistically significant (*Z* = 0.89, *p* = 0.374) (all *p* > 0.05) (Table 3).

**TABLE 3:** Comparisons of demographic and clinical characteristics between the SR-P and NSR-P groups.

Variables	NSR-P (*N* = 88)	SR-P (*N* = 80)	Statistic (*t* or *x*^2^or z)	OR	95% CI		*P*-value
					Lower	Upper	
Age (years), mean (SD)^a^	34.60 (12.73)	25.79 (10.51)	4.91	1.070	1.037	1.104	<0.001*
Sex, *n* (%)			4.36	0.507	0.267	0.963	0.037*
Male	39 (44.31)	23 (28.75)					
Female	49 (55.68)	57 (71.25)					
Education, *n* (%)			0.30	1.211	0.611	2.398	0.584
High school and below (<12 years)	22 (25.00)	23 (28.75)					
Above high school (≥12 years)	66 (75.00)	57 (71.25)					
Marital status, *n* (%)			11.35	3.068	1.581	5.955	0.001*
Married	43 (48.86)	19 (23.75)					
Unmarried	45 (51.14)	61 (76.25)					
Vocational status, *n* (%)			0.31	0.794	0.350	1.800	0.580
On the job	72 (81.82)	68 (85.00)					
Unemployment	16 (18.18)	12 (15.00)					
Duration of illness (months), median^b^	6.00	6.50	0.89	–	–	–	0.374
HAMD total scores, mean (SD)^a^	20.83 (6.42)	25.61 (6.61)	5.23	0.873	0.823	0.925	<0.001*
HAMD scores without item 3, mean (SD)^a,c^	19.89 (5.02)	24.00 (6.42)	4.60	0.883	0.833	0.936	<0.001*

*SR-P, suicidal risk MDD patient; NSR-P, non-suicidal risk MDD patient.*

*Statistical significance.

*^a^t-tests.*

*^b^Rank-sum tests.*

*^c^Third (suicide) item was removed from the total Hamilton Depression Rating Scale (HAMD) score.*

**FIGURE 1:**
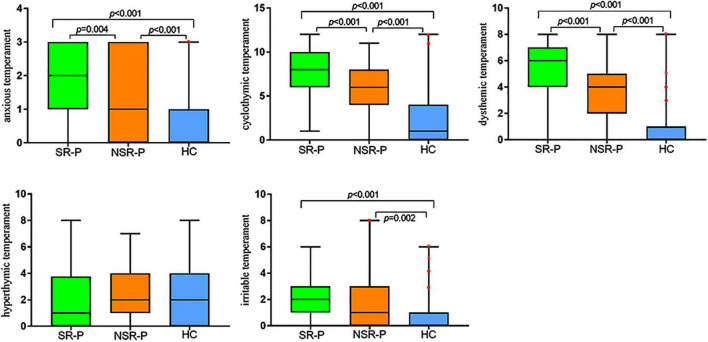
Box plots of the TEMPS-A subscales across the groups of SR-P, NSR-P and HC. The solid lines show the medians, and the boxes show the interquartile ranges. Outlier (•) means cases with values between 1.5 and 3 box lengths from the upper or lower edge of the box. Asterisk (*) means cases with values more than 3 box lengths from the upper or lower edge of the box. The box length is the interquartile range. SR-P: suicidal risk MDD patients; NSR-P: non-suicidal risk MDD patients; and HC: healthy control. *P*-value is adjusted by Bonferroni correction.

Upon comparing the differences in temperament scores in SR-P, NSR-P, and HCs subgroups in all five affective temperaments, the distribution of scores was not the same (Figure 1). Scores for cyclothymic temperament (*H* = 121.932, *p* < 0.001), dysthymic temperament (*H* = 150.393, *p* < 0.001), irritable temperament (*H* = 35.430, *p* < 0.001), and anxious temperament (*H* = 92.437, *p* < 0.001) were significantly different. The median scores for dysthymic temperament were 6 (*n* = 80) in the SR-P group, 4 (*n* = 88) in the NSR-P group, and 0 (*n* = 116) in the HCs group. The median score of the whole was 3 (*n* = 284). When the Bonferroni correction was performed on the statistical significance level of the pairwise comparison, the distributions of dysthymic, cyclothymic, and anxious temperament scores between three subgroups were all statistically significant (all *p* < 0.005) (Figure 1), and irritable temperament showed no significant difference between the SR-P and the NSR-P subgroups (*Z* = 2.381, adjusted *p* = 0.052) (Table 4). The differences among the three subgroups on the hyperthymic temperament were not statistically significant (*H* = 4.59, *p* = 0.101). The scoring directions of the three subgroups on temperaments were all suicidal risk group >non-suicidal risk group >healthy control group, except hyperthymic temperament (Table 4 and Figure 1).

**TABLE 4:** Comparisons of TEMPS-A subscales among the SR-P, NSR-P, and HC groups.

	Statistic z			*Post-hoc* tests adj. sig.			Mean rank	Median	Statistic H	*P*-value
	SR-P	NSR-P	HC	SR-P	NSR-P	HC				
**Cyclothymic**								5	121.93	<0.001*
SR	–	3.93	10.76	–	<0.001*	<0.001*	210.02	8		
NSR		–	6.77		–	<0.001*	160.49	6		
HC			–			–	62.29	1		
**Dysthymic**								3	150.39	<0.001*
SR	–	3.53	11.72	–	<0.001*	<0.001*	212.61	6		
NSR		–	8.19		–	<0.001*	168.36	4		
HC			–			–	74.53	0		
**Hyperthymic**								2	4.59	0.101
SR	–	–	–	–	–	–	–	1		
NSR	–	–	–	–	–	–	–	2		
HC	–	–	–	–	–	–	–	2		
**Irritable**								1	35.43	<0.001*
SR	–	2.38	5.86	–	0.052	<0.001*	178.89	2		
NSR		–	3.42		–	0.002*	149.90	1		
HC			–			–	111.79	0		
**Anxious**								1	92.44	<0.001*
SR	–	3.22	9.32	–	0.004*	<0.001*	198.05	2		
NSR		–	6.06		–	< 0.001*	158.99	1		
HC			–			–	91.68	0		

*SR-P, suicidal risk MDD patients; NSR-P, non-suicidal risk MDD patients; and HC, healthy control.*

*Difference among three groups, Kruskal–Wallis test, p < 0.001, Bonferroni correction: 0.05/5 tests = 0.01.

### Multivariable Logistic Regression Modeling

Factors preliminarily associated with suicidal risk among those with depressive disorders were further tested by multivariable logistic regression modeling. The HAMD scale includes suicide items, so we subtracted the item-3 score from the HAMD summary score. The logistic regression modeling was statistically significant (*x*^2^ = 72.846, *p* < 0.001). The final factors independently associated with suicide risk were current age (*OR* = 0.937, 95% *CI* 0.905∼0.970), sex (*OR* = 2.606, 95% *CI* 1.142∼5.948), HAMD score without the third item (*OR* = 1.145, 95% *CI* 1.063∼1.234), and cyclothymic (*OR* = 1.275, 95% *CI* 1.102∼1.475), and dysthymic temperaments (*OR* = 1.265, 95% *CI* 1.037∼1.542) (Table 5).

**TABLE 5:** Factors associated with suicidal risk in MDD: multivariate logistic regression analysis.

Variates	β	OR	95% CI		*P*-value
			Lower	Upper	
Age	−0.065	0.937	0.905	0.970	<0.001
Sex (female/male)	0.958	2.606	1.142	5.948	0.023
HAMD score without the 3rd item^a^	0.136	1.145	1.063	1.234	<0.001
Cyclothymic temperament	0.243	1.275	1.102	1.475	0.001
Dysthymic temperament	0.235	1.265	1.037	1.542	0.020

*^a^Third (suicide) item was removed from the total HAMD score.*

## Discussion

Biopsychosocial factors converge in suicide is allostasis, as an adaptive physiological response occurs when an organism is challenged. There were some hypothesizes on the converge relations. Previous studies indicate that cyclothymic and anxious temperament traits (assessed with the TEMPS-A) are associated with higher stress (29). Stress alters neuroplasticity in humans assessed with transcranial magnetic stimulation, and altered neuroplasticity has been associated with worsening of depression (30, 31). Indeed, indicators of stress (increased allostatic load) have been associated with suicide (32). The convergent relations include all the pathological mechanism and social and psychological factors.

To the best of our knowledge, this is the first study exploring the associations of temperament, depressive symptoms, and suicidal risk among patients with first-episode of MDD. In these first-onset MDD subjects, we obtained the following: (1) suicide rate; (2) temperament trait differences from HCs; (3) demographic characteristics and temperament differences between the suicidal and non-suicidal groups; and (4) cyclothymic and dysthymic temperament, as well as sex, age, and severe depression, were independent suicidal risk factors.

Many studies have shown that depressive episode frequency is closely related to suicidal risk (8, 33, 34). A higher episode frequency means a higher suicide rate. In this study, we found that the suicide rate of first-episode of MDD was as high as 47.62%, close to previous studies of MDD from 47 to 69% (35–37), meaning that nearly half of the patients with depression had suicidal ideation or attempted suicide during their first major depressive episode, so we should pay attention to the suicide risk from the very first episode of MDD.

Approximately 20% of the general population has a certain type of emotional temperament (13). In this study, the mean rank direction of temperaments in HCs was hyperthymic > irritable > anxious > cyclothymic > dysthymic, and in MDD was dysthymic > cyclothymic > anxious > irritable > hyperthymic. These directions showed the different temperament tendencies in different population. The two exactly opposite directions in two populations is interesting and needs further research. This study tried to identify suicide risk factors in patients with first-episode depression. Compared with HCs, patients with first-episode MDD scored significantly different on dysthymic and cyclothymic temperaments. Generally, women are prone to have dysthymic, cyclothymic, and anxious temperament, while men are prone to have hyperthymic and irritable temperament (15). In our study, the mean rank direction of temperament in MDD also showed that the dysthymic outnumbered hyperthymic temperament, which also proved to be the truth that women were more prone to being depressed than men.

We compared the temperament differences between suicidal and non-suicidal risk in patients with depression. As expected, cyclothymic and dysthymic temperaments were associated with a greater risk of suicide. The five factors significantly and independently differentiated patients with vs. without suicidal risk based on multivariable logistic regression modeling are as follows: female sex, current age, dysthymic temperament, cyclothymic temperament, and higher HAMD score. The five factors set a model of high suicide risk. The final factors/features of suicide-risk patients were the same as the clinical impressions. People with cyclothymic temperament traits show instability in mood, thoughts, and behaviors, while mood instability usually leads to suicidal behavior, and quickly changing mood stations enhance suicidal ideation and attempts (15). Depressive patients with cyclothymic temperament traits are probably developing bipolar disorder (38), as well as high rates of suicide and hospitalization (21, 39). All the bad outcomes make the early recognition of MDD and suicide risk necessary.

Many previous studies have examined the demographic characteristics of suicide risk factors in patients with depressive disorder. As expected, the onset age was strongly associated with suicide, which was also supported by other studies (40). Early onset age (especially <18 years) of depression is associated with an increasing rate of conversion to bipolar disorder (41), which is also a highly risk factor for suicide (21, 42). Subjects of this study were people with a current age over 18 years, and the mean illness duration was 6–6.5 months; we did not obtain the suicide rate of younger adolescents, which still needs further research. Other demographic suicide risk factors in MDD included female sex (71.25%) and unmarried status (76.25%), which were in accordance with previous observations (21, 43). We found that a greater suicide risk was more associated with female sex, which was also confirmed in other studies (16, 44). However, men may have more violent suicide attempts than women, and lethal action makes their suicide death rate higher than in women (42), which also needs further study. Our study showed that unmarried and unemployed people are easily prone to depression, and unmarried people have significantly higher instances of thoughts or actions related to suicide.

In our study, first-episode depression was detected by HAMD. We found that HAMD had removed the third (suicide) item score, which could still predict suicidal risk in MDDs. This means, in addition to the third (suicide) item, all the other HAMD-17 items’ total score is closely linked to suicide risk (45, 46). Particularly of note, the standardized HAMD scale predicting suicide may result in approximately 30% false positives (47), so it is risky to take HAMD score as a sole predict factor of suicide risk. The best chance to recognize suicide risk would be to confirm predictors as much as we can.

After confirming the predictors of suicidal risk, especially the temperament factor, could suicide be intervened? Serotonin is a neurotransmitter involved in many brain and body functions. Impaired serotonergic function is involved in the development of suicide behavior and was proven decades ago (48). Some studies on genes showed that depression and suicide were at the same gene locus (6, 49–51), other than a causal relationship. They found that the S allele in the serotonin transporter-linked polymorphic region (5-HTTLPR) may be associated with affective temperament, especially cyclothymic temperament (52). Sarmiento-Hernández’s study showed that the S-allele and the SS genotypes of 5-HTTLPR were associated with suicide attempts (53). They provide a theoretical basis for the relationship between cyclothymic temperament and suicide. If the suicide risk and temperament are genetic phenotypes and share the same locus, is intervention difficult? Other studies have shown that affective temperaments may change to some degree, and people could exhibit one type of character trait during puberty and deviate when they grow up (54). They provide the basis that temperament is changeable throughout a person’s life (55). Japanese researcher Inoue studied the influence of ambient temperature on temperament (56), which makes us believe that the final outcome could be intervened.

## Limitations

Several important limitations should be considered. First, our study had a cross-sectional design, and the factors associated with suicide should not be considered direct risk factors or “causes.” Second, the self-report scale may cause recall bias. Third, the single site of observation may cause selection bias, and some high suicide risk patients did not ask doctors for help. Fourth, due to cultural background differences, the results may not be suitable for other populations or ethnicities. Finally, there were few analyzable factors in our study. More factors would make the results more convincing.

## Conclusion

The findings of the study support the notion that there are temperament differences between the general population and patients with depression. People with cyclothymic or dysthymic temperament traits have a higher suicide risk than those with other temperament traits in patients with first-onset MDD. Young women with cyclothymic or dysthymic temperaments may be the highest suicidal risk population.

## Data Availability Statement

The raw data supporting the conclusions of this article will be made available by the authors, without undue reservation.

## Ethics Statement

The studies involving human participants were reviewed and approved by the Ethics Committee of Beijing HuiLongGuan Hospital. The patients/participants provided their written informed consent to participate in this study.

## Author Contributions

LY: conceptualization, data curation, formal analysis, and writing the original draft. T-HS: conceptualization, methodology, validation, investigation, and writing the original draft. Y-YW and L-GZ: funding acquisition, conceptualization, supervision, and validation. S-JZ: investigation, methodology, supervision, and validation. J-JY and L-YZ: investigation, methodology, and supervision. H-JL: investigation and methodology. J-XC: study design. All authors contributed to the article and approved the submitted version.

## Conflict of Interest

The authors declare that the research was conducted in the absence of any commercial or financial relationships that could be construed as a potential conflict of interest.

## Publisher’s Note

All claims expressed in this article are solely those of the authors and do not necessarily represent those of their affiliated organizations, or those of the publisher, the editors and the reviewers. Any product that may be evaluated in this article, or claim that may be made by its manufacturer, is not guaranteed or endorsed by the publisher.
